# Supplementary material for: Soil Microbial Indicators within Rotations and Tillage Systems
Source: Microorganisms. 2021 Jun 8;9(6):1244. doi: 10.3390/microorganisms9061244 (PMC8228827; doi:10.3390/microorganisms9061244)
Supplement: Supplementary file 1 [file microorganisms-09-01244-s001.zip › microorganisms-1247538-supplementary.pdf]

# Soil microbial indicators within rotations and tillage systems

**Gevan D Behnke[1], Nakian Kim[1], Maria C Zabaloy[2], Chance Riggins[1], Sandra Rodriguez-Zas[3] and Maria B. Villamil[1*]**

[1]University of Illinois, Crop Sciences, Urbana, IL, USA

[2] Centro de Recursos Naturales Renovables de la Zona Semiárida (CERZOS, UNS-CONICET), Departamento de Agronomía, Universidad Nacional del Sur, Bahia Blanca, Argentina

[3]University of Illinois, Animal Sciences, Urbana, IL, USA

*** Correspondence:**
Villamil@illinois.edu

**Supplementary Material**

**Table S1.** Summary of field practices from Monmouth, Illinois from 1997-2016; dates are ranges that vary slightly year to year.

| Field event type | Dates |
|---|---|
| Tilled wheat plots | Late Sept - early Oct |
| Planted wheat crop | Late Sept - early Oct |
| Fall wheat fertilization | Late Oct - early Nov |
| Fall tillage of corn and soybean plots† | Mid Nov |
| Spring wheat fertilization | Late March - early Apr |
| Secondary tillage in corn and soybean plots‡ | Mid April - early May |
| Spring corn fertilization | Mid April - mid May |
| Corn planting | Mid April - mid May |
| Soybean planting | Early - mid May |
| Harvest of wheat | Late June - late July |
| Harvest of corn | Late Sept - early Oct |
| Harvest of soybean | Late Sept - early Oct |

†Chisel tillage used a disk-ripper 14" deep in plots designated as tilled; no till received zero tillage
‡Secondary tillage used a field cultivator in plots designated as tilled; no-till received zero tillage

**Table S2.** Principal component analysis of soil variables (17) for 0-10 cm soil depth with eigenvalues and cumulative proportion of the data set variability explained by the five principal components (PC) extracted with eigenvalues >1. Component correlation scores (eigenvectors) with loadings greater than |0.5| are bolded. Probability values for the analysis of variance (ANOVA) and degrees of freedom (df) available for the effects of rotation|phase (Rotation), tillage (Tillage), and their interaction are shown for each extracted PCs.

| | | **PC1** | **PC2** | **PC3** | **PC4** | **PC5** |
|---|---|---|---|---|---|---|
| **Eigenvalue** | | 4.8 | 4.07 | 1.87 | 1.32 | 1.2 |
| **Cum. Proportion** | | 0.28 | 0.52 | 0.63 | 0.71 | 0.78 |
| **Soil variable** | | **Component Correlation Scores** | | | | |
| pH | | **0.76** | **-0.6** | -0.06 | 0.02 | 0.09 |
| CEC | | -0.11 | **0.95** | -0.04 | -0.08 | -0.11 |
| SOM | | -0.23 | **0.59** | **-0.61** | -0.01 | 0.21 |
| $NO_3^-$ | | 0.27 | 0.16 | **0.66** | -0.4 | -0.17 |
| $NH_4^+$ | | -0.38 | 0.15 | -0.26 | **0.65** | 0.08 |
| P | | 0.31 | 0.41 | 0.25 | 0.39 | 0.35 |
| S | | -0.32 | 0.12 | 0.13 | **-0.55** | **0.55** |
| K | | **-0.55** | 0.25 | 0.34 | 0.04 | 0.23 |
| Na | | **0.55** | **0.71** | -0.14 | -0.07 | -0.16 |
| Ca | | **0.87** | 0.35 | -0.06 | 0.12 | -0.14 |
| Mg | | -0.05 | 0.28 | 0.39 | 0.1 | **-0.58** |
| Al | | **-0.74** | 0.08 | **0.49** | 0.29 | -0.06 |
| Fe | | **-0.49** | **0.77** | 0.2 | 0.1 | 0.01 |
| B | | **0.7** | 0.09 | 0.28 | 0.15 | 0.33 |
| Zn | | 0.44 | **0.71** | 0.18 | -0.06 | 0.29 |
| Cu | | **0.77** | 0.45 | -0.17 | -0.02 | -0.11 |
| Mn | | **0.56** | -0.37 | 0.43 | 0.36 | 0.2 |
| **Factor** | **df** | | | **Probability values** | | |
| Rotation | 3 | *0.0061* | *0.0175* | *0.0638* | *0.0005* | *0.0574* |
| Tillage | 1 | *0.0012* | *0.0006* | *<.0001* | 0.5677 | *0.0367* |
| Rotation x Tillage | 3 | 0.8511 | 0.8368 | 0.223 | 0.7103 | 0.6221 |

**Table S3.** List of bacterial principal components (PC) comprised by indicator species that contributed at least 5% of the variability in the data and with eigenvalue of at least 1.

| PC | Eigenvalue | Proportion (%) | Loadings | Phylum | Class | Order | Family | Genus | Species |
|---|---|---|---|---|---|---|---|---|---|
| 1 | 12.76 | 36% | -0.76 | Acidobacteria | Acidobacteriia | Acidobacteriales | Acidobacteriaceae (Subgroup 1) | | |
| | | | -0.76 | Acidobacteria | Acidobacteriia | Solibacterales | Solibacteraceae (Subgroup 3) | *Candidatus* | *solibacter* |
| | | | 0.63 | Acidobacteria | Acidobacteriia | Solibacterales | Solibacteraceae (Subgroup 3) | *Paludibaculum* | uncultured bacterium |
| | | | 0.78 | Acidobacteria | Blastocatellia (Subgroup 4) | Pyrinomonadales | Pyrinomonadaceae | *RB41* | uncultured bacterium 16H1 |
| | | | 0.80 | Acidobacteria | Holophagae | Subgroup 7 | | | |
| | | | 0.60 | Acidobacteria | Subgroup 25 | uncultured bacterium | uncultured bacterium | uncultured bacterium | uncultured bacterium |
| | | | 0.77 | Actinobacteria | Acidimicrobiia | Actinomarinales | uncultured | uncultured soil bacterium | uncultured soil bacterium |
| | | | -0.50 | Actinobacteria | Actinobacteria | Micrococcales | Microbacteriaceae | | |
| | | | 0.69 | Actinobacteria | MB-A2-108 | Actinomycetales | uncultured Actinomycetales bacterium | uncultured Actinomycetales bacterium | uncultured Actinomycetales bacterium |
| | | | 0.58 | Actinobacteria | MB-A2-108 | Actinobacterium | uncultured actinobacterium | uncultured actinobacterium | uncultured actinobacterium |
| | | | 0.57 | Chloroflexi | Anaerolineae | RBG-13-54-9 | uncultured bacterium UASB_TL14 | uncultured bacterium UASB_TL14 | uncultured bacterium UASB_TL14 |
| | | | 0.75 | Chloroflexi | Dehalococcoidia | S085 | uncultured bacterium | uncultured bacterium | uncultured bacterium |
| | | | -0.83 | Chloroflexi | Ktedonobacteria | Ktedonobacterales | JG30-KF-AS9 | uncultured bacterium | uncultured bacterium |
| | | | 0.66 | Planctomycetes | OM190 | uncultured bacterium | uncultured bacterium | uncultured bacterium | uncultured bacterium |
| | | | -0.87 | Planctomycetes | Phycisphaerae | Tepidisphaerales | WD2101 soil group | uncultured Planctomycetales bacterium | uncultured Planctomycetales bacterium |
| | | | 0.55 | Planctomycetes | Phycisphaerae | mle1-8 | uncultured bacterium | uncultured bacterium | uncultured bacterium |
| | | | -0.88 | Proteobacteria | Alphaproteobacteria | Micropepsales | Micropepsaceae | uncultured | |
| | | | 0.80 | Proteobacteria | Alphaproteobacteria | Rhizobiales | Rhizobiales Incertae Sedis | *Nordella* | |
| | | | 0.67 | Proteobacteria | Gammaproteobacteria | Betaproteobacteriales | TRA3-20 | | |
| | | | -0.70 | Proteobacteria | Gammaproteobacteria | Xanthomonadales | Rhodanobacteraceae | *Chujaibacter* | |
| | | | -0.57 | Proteobacteria | Gammaproteobacteria | Xanthomonadales | Rhodanobacteraceae | *Rhodanobacter* | |
| | | | 0.54 | Rokubacteria | NC10 | Rokubacteriales | uncultured Firmicutes bacterium | uncultured Firmicutes bacterium | uncultured Firmicutes bacterium |
| | | | -0.83 | Verrucomicrobia | Verrucomicrobiae | Pedosphaerales | Pedosphaeraceae | bacterium Ellin516 | bacterium Ellin516 |
| 2 | 3.12 | 9% | -0.73 | Bacteroidetes | Bacteroidia | Chitinophagales | | | |
| | | | -0.60 | Proteobacteria | Gammaproteobacteria | Betaproteobacteriales | Nitrosomonadaceae | *MND1* | uncultured Nitrosomonadales bacterium |
| 3 | 2.64 | 8% | -0.64 | Actinobacteria | Thermoleophilia | Gaiellales | Gaiellaceae | *Gaiella* | |
| | | | 0.60 | Bacteroidetes | Bacteroidia | Sphingobacteriales | AKYH767 | metagenome | metagenome |
| | | | 0.63 | Proteobacteria | Gammaproteobacteria | R7C24 | metagenome | metagenome | metagenome |
| 4 | 2.17 | 6% | 0.49 | Proteobacteria | Deltaproteobacteria | Myxococcales | Archangiaceae | | |
| 5 | 2.06 | 6% | -0.63 | Proteobacteria | Gammaproteobacteria | Xanthomonadales | Xanthomonadaceae | *Luteimonas* | |

**Table S4.** List of fungal principal components (PC) comprised by indicator species that contributed at least 5% of the variability in the data and with eigenvalue of at least 1.

| PC | Eigenvalue | Proportion (%) | Loadings | Phylum | Class | Order | Family | Genus | Species |
|---|---|---|---|---|---|---|---|---|---|
| 1 | 3.06 | 8% | 0.55 | Ascomycota | Sordariomycetes | Hypocreales | Nectriaceae | *Fusarium* | *sporotrichioides* |
| | | | 0.53 | Ascomycota | Dothideomycetes | Capnodiales | Mycosphaerellaceae | | |
| 2 | 2.58 | 7% | 0.58 | Ascomycota | Sordariomycetes | Glomerellales | Plectosphaerellaceae | *Gibellulopsis* | *piscis* |
| | | | 0.71 | Ascomycota | Sordariomycetes | Glomerellales | Plectosphaerellaceae | *Plectosphaerella* | |
| | | | 0.58 | Ascomycota | Sordariomycetes | Coniochaetales | Coniochaetaceae | | |
| 3 | 2.51 | 7% | 0.73 | Ascomycota | Sordariomycetes | Sordariales | Lasiosphaeriaceae | *Schizothecium* | |
| | | | 0.56 | Ascomycota | Sordariomycetes | Sordariales | Lasiosphaeriaceae | *Schizothecium* | *carpinicola* |
| 4 | 2.12 | 6% | 0.51 | Ascomycota | Sordariomycetes | Hypocreales | Bionectriaceae | *Clonostachys* | *rosea* |
| 5 | 2.08 | 6% | 0.49 | None selected | | | | | |

**Table S5.** List of archaeal principal components (PC) comprised by indicator species that contributed at least 5% of the variability in the data and with eigenvalue of at least 1.

| PC | Eigenvalue | Proportion (%) | Loadings | Phylum | Class | Order | Family | Genus | Species |
|---|---|---|---|---|---|---|---|---|---|
| 1 | 2.24 | 20% | 0.54 | Thaumarchaeota | Nitrososphaeria | Nitrososphaerales | Nitrososphaeraceae | *Candidatus* | *nitrocosmicus* |
| | | | 0.50 | Thaumarchaeota | Nitrososphaeria | Nitrososphaerales | Nitrososphaeraceae | uncultured ammonia-oxidizing archaeon | uncultured ammonia-oxidizing archaeon |
| | | | -0.56 | Thaumarchaeota | Nitrososphaeria | Nitrososphaerales | Nitrososphaeraceae | uncultured soil archaeon | uncultured soil archaeon |
| | | | 0.60 | Thaumarchaeota | Nitrososphaeria | Nitrososphaerales | Nitrososphaeraceae | | |
| | | | -0.84 | Thaumarchaeota | Nitrososphaeria | Nitrosotaleales | Nitrosotaleaceae | *Candidatus* | *nitrosotalea* uncultured archaeon |
| 2 | 1.69 | 15% | -0.29 | Thaumarchaeota | Nitrososphaeria | Nitrososphaerales | Nitrososphaeraceae | uncultured compost archaeon | uncultured compost archaeon |
| | | | -0.72 | Thaumarchaeota | Nitrososphaeria | Nitrososphaerales | Nitrososphaeraceae | | |
| 3 | 1.27 | 12% | 0.72 | Nanoarchaeaeota | Woesearchaeia | | | | |
| 4 | 1.26 | 11% | 0.75 | Euryarchaeota | Thermoplasmata | Marine Group II | uncultured archaeon | uncultured archaeon | uncultured archaeon |
| 5 | 1.08 | 10% | 0.59 | Euryarchaeota | Thermoplasmata | uncultured | uncultured archaeon | uncultured archaeon | uncultured archaeon |
| | | | -0.57 | Thaumarchaeota | Nitrososphaeria | Nitrososphaerales | Nitrososphaeraceae | *Candidatus* | *mitrososphaera* |

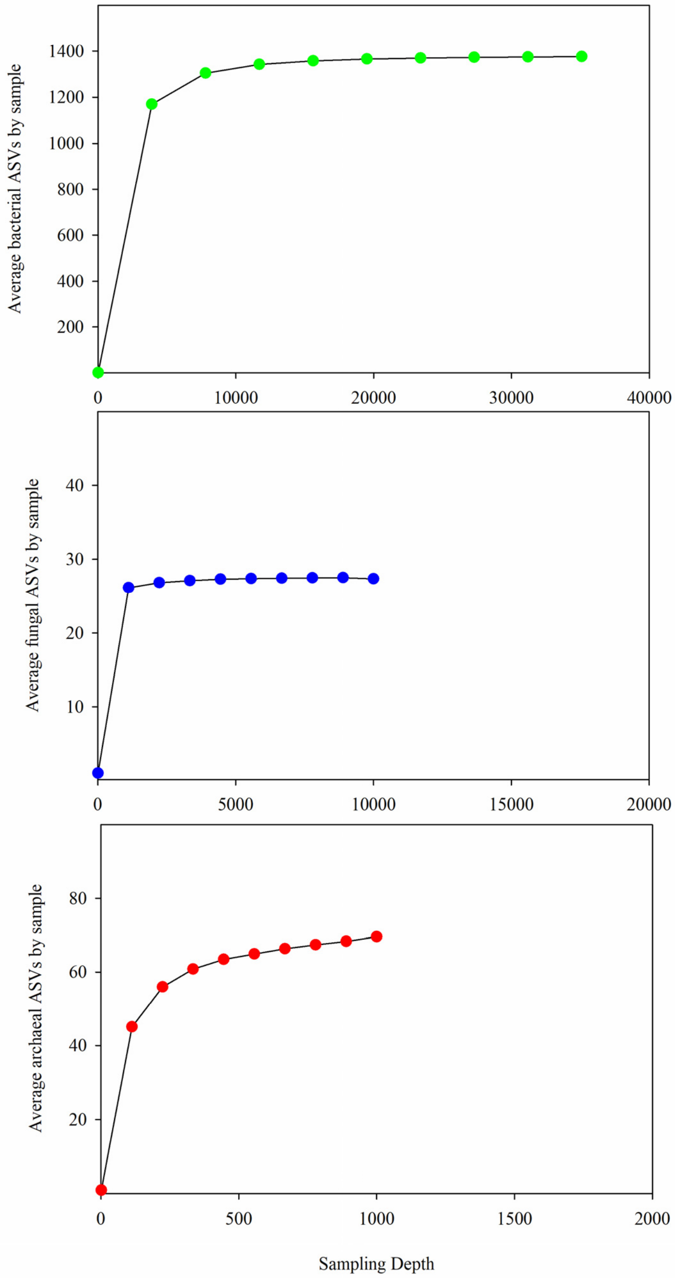


**Figure. S1**. Rarefaction curves for the major kingdoms of bacteria (green; top panel), fungi (blue; middle panel), and archaea (red; bottom panel). Points show the average number of observed ASVs for a given sampling depth (x-axis). For each taxa, rarefaction curves plateaued at sampling depths of 35,100, 10,000, and 1000 for bacterial, fungal, and archaeal sequences per sample, respectively.
